# Spontaneous tension pneumothorax as a complication of Coronavirus disease 2019: Case report and literature review

**DOI:** 10.1002/ccr3.5852

**Published:** 2022-05-09

**Authors:** Fateen Ata, Zohaib Yousaf, Rana Farsakoury, Adeel Ahmad Khan, Abdullah Arshad, Maya Omran, Dore Chikkahanasoge Ananthegowda, Mohamad Khatib, Talat Saeed Chughtai

**Affiliations:** ^1^ Department of Internal Medicine Hamad General Hospital Hamad Medical Corporation Doha Qatar; ^2^ Department of Plastic Surgery Hamad General Hospital Hamad Medical Corporation Doha Qatar; ^3^ Department of Endocrinology Hamad General Hospital Hamad Medical Corporation Doha Qatar; ^4^ Medical Intensive Care Unit Hazm Mebaireek Hospital Hamad Medical Corporation Doha Qatar; ^5^ Trauma Surgery and Thoracic Surgery Hamad General Hospital Hamad Medical Corporation Doha Qatar

**Keywords:** coronavirus disease 2019, COVID‐19, SARS‐CoV‐2, severe acute respiratory syndrome coronavirus 2, spontaneous pneumothorax, tension pneumothorax

## Abstract

Primary spontaneous tension pneumothorax (STP) is a rare and life‐threatening condition. We report a case of COVID‐19‐pneumonia patient who developed STP as a complication. He had a prolonged hospital stay and was ultimately discharged asymptomatic. A systematic literature search was performed to review studies (*N*=12) reporting STP in the setting of COVID‐19.

## Graphical abstract

Tension pneumothorax can present such as COVID‐19 infection, leading to a delayed diagnosis, and may increase mortality. In the absence of risk factors, COVID‐19 can be an independent risk factor for STP development with significant mortality rates.

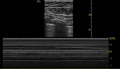

## 1 INTRODUCTION

The SARS‐CoV‐2 virus continues to be a public health emergency due to the ongoing pandemic, which is now entering its third year. As per the latest World Health Organization (WHO) situation report, by January 11, 2022, above 300 million people have been infected by the SARS‐CoV‐2 virus since the start of the pandemic, with more than 5.4 million fatalities.^1^ COVID‐19 is primarily a respiratory disease. The pulmonary complications are a sequela of parenchymal, vascular, or pleural involvement and range from pneumonia to acute respiratory distress syndrome (ARDS) and pulmonary embolism to pneumothorax.^2^ Pneumothorax, defined as air within the pleural space, is one of the emerging complications of COVID‐19 infection.^3^ It can be primary (without any underlying lung disease) or secondary to chronic lung disease. If the air enters the pleural cavity but cannot leave, hence creating a one‐way valve, and leads to mediastinal shift and associated decrease in venous return, cardiac output, and ultimately hypotension; then, it is referred to as tension pneumothorax, which is fatal, if left untreated.^4^ Multiple cases of COVID‐19 infection complicated with the development of STP have been reported till date.^5^, ^6^, ^7^, ^8^, ^9^, ^10^, ^11^, ^12^, ^13^, ^14^, ^15^, ^16^ This emerging complication of COVID‐19 infection seems to have significant mortality. In addition, the diagnosis can be challenging given that COVID‐19 infection may mask the symptoms of STP, as both may have similar symptomatology. This case report and detailed literature review highlight this severe complication of COVID‐19, providing relevant insights to the physicians managing COVID‐19 cases amid an ongoing pandemic.

## 2 CASE PRESENTATION

**FIGURE 1:**
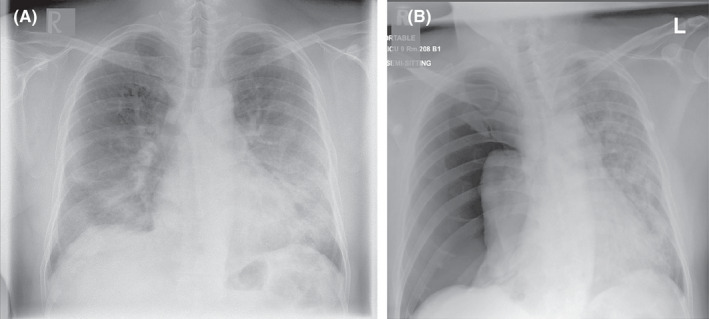
(A) Chest X‐ray on presentation with COVID‐19 pneumonia showing bilateral middle and lower zone infiltrates. (B) Chest X‐ray (day 22) postero‐anterior view showing development of right‐sided tension pneumothorax with collapsed right lung and pushed heart and mediastinum to the left side

A 40‐year‐old Filipino gentleman presented with a two‐day history of sore throat and non‐exertional shortness of breath. There was no history of fever or chest pain. The patient did not have any history of sick contacts, recent travel, immobilization, or recent surgery. He did not have any chronic medical illnesses, was not taking any medication, and was a non‐smoker. On physical examination, he had a temperature of 36.5°C, blood pressure of 130/82 mmHg, and respiratory rate (RR) of 36 breaths/min. He was desaturating on room air to 82% and required 15 liters of oxygen via a non‐rebreather mask to maintain oxygen saturation above 95%. The patient had pharyngeal erythema and bilateral coarse crackles on respiratory examination. Cardiac, gastrointestinal, and neurological examination were unremarkable. Complete blood count revealed lymphopenia but normal total white cell count, hemoglobin, and platelet counts. The metabolic profile showed hyponatremia, hypokalemia, elevated alanine aminotransferase (ALT), aspartate aminotransferase (AST), and lactate dehydrogenase. He also had an elevated C‐reactive protein (CRP) and interleukin‐6 (IL‐6), but a normal d‐dimer level (Table 1). A chest X‐ray showed bilateral middle and lower zone infiltrates (Figure 1A). A reverse‐transcriptase polymerase chain reaction (RT‐PCR) nasopharyngeal swab was positive for SARS‐CoV‐2. The rest of the respiratory viral and blood cultures was negative for any other infective organism. Genomic testing was not performed to identify the strain of SARS‐CoV‐2. However, at the time of the patient's presentation, the Delta variant (B.1.617.2) was prevalent in Qatar.

**TABLE 1:** Laboratory parameters of the patient at admission

Parameter (normal range)	Results
White cells (4–10 × 10^3^/mm^3^)	5.9
Neutrophil (2–7*10^3^/mm^3^)	4.8
Lymphocyte (1–3 × 10^3^/mm^3^)	0.7
Platelets (150–400 × 10^3^/mm^3^)	166
Hemoglobin (12.5–13.5 gm/dl)	13.6
Red Blood cells (4.5–5.5 10^3^/mm^3^)	4.7
ALT (0–55 Unit/L)	269
AST (5–34 Unit/L)	87
Creatinine (62–106 umol/L)	81
Sodium (mmol/L)	128
Potassium (mmol/L)	3.3
D‐dimer (<mg/L FEU)	0.36
CRP (0–5 mg/L)	83.1
Procalcitonin (<0.5 ng/ml)	0.36
Ferritin (48–420 µgm/L)	1031
Interleukin−6 (≤7 pg/ml)	37

Abbreviations: ALT, alanine aminotransferase; AST, aspartate aminotransferase; CRP, C‐reactive protein.

The patient was admitted to the intensive care unit as a case of severe COVID‐19 pneumonia. As per the institutional protocol at the time, he was treated with intravenous (IV) ceftriaxone, azithromycin, methylprednisolone (40 mg every 12 hourly), and IV Remdesivir. Despite treatment, the patient's condition did not improve, and on Day 5, intravenous immunoglobulin (IVIG) 0.4gmlkg/day for three doses was initiated. On Days 6 and 7 of admission, he received two doses of IV tocilizumab 800mg. On Days 8 and 9, the patient received four units of convalescent plasma. The patient received multidisciplinary care throughout his admission, including physiotherapy, occupational therapy, respiratory therapy, dietetics, critical care, and infectious disease. On Day 14 of hospital stay, due to persistently high oxygen requirements and increasing D‐dimers (peak 7.2 mg/L FEU), a CT pulmonary angiogram (CTPA) was performed to rule out pulmonary embolism. The scan showed no filling defect but showed predominantly peripheral and patchy basal areas of ground‐glass attenuation with multifocal segmental dense consolidation with air bronchograms, consistent with severe bilateral pneumonia due to COVID‐19.

**FIGURE 2:**
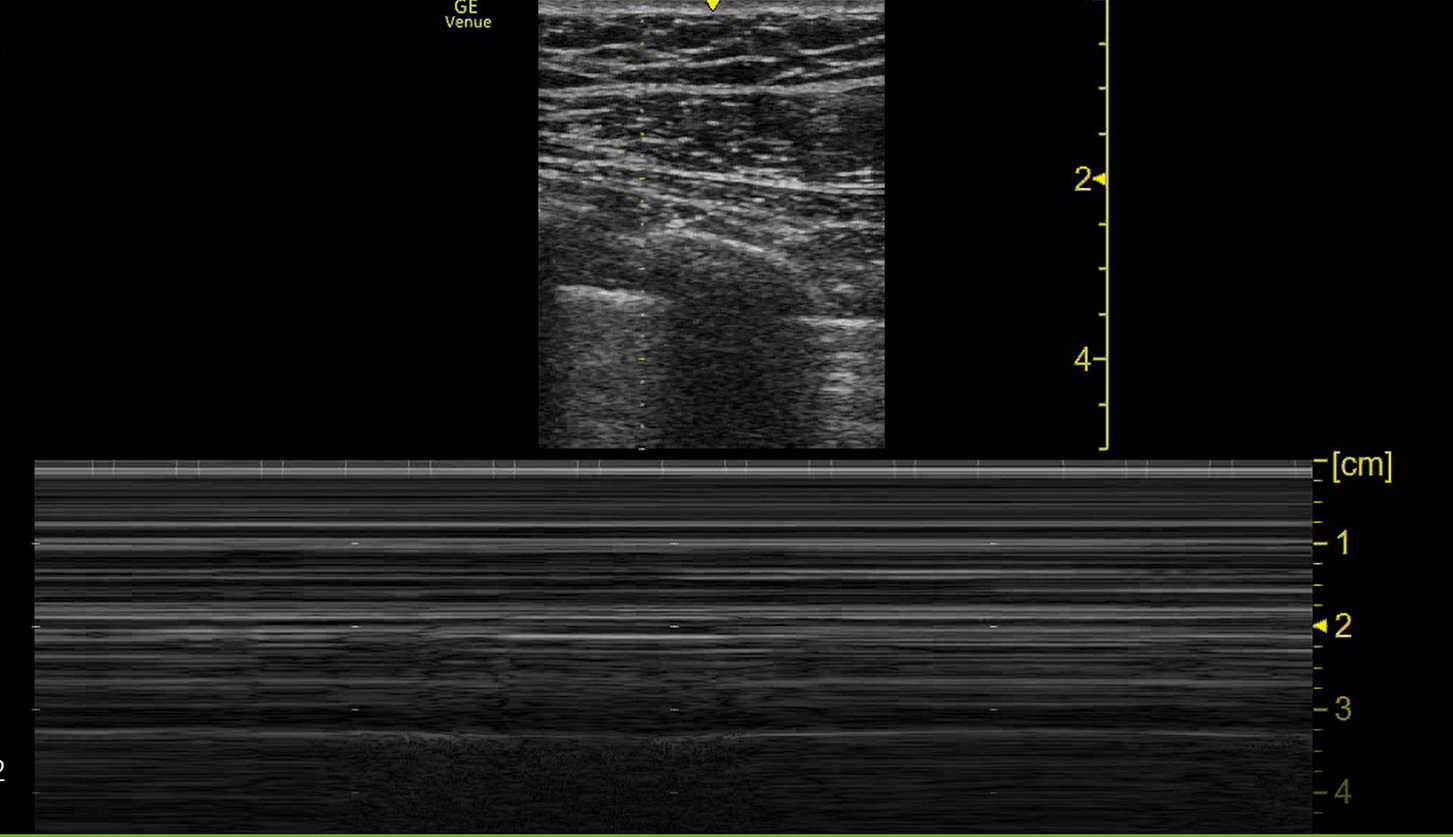
US pleural cavity showing bar‐code sign consistent with pneumothorax

After a period of stability, on Day 22 of hospitalization, the patient developed sudden tachypnea (41 breaths/min), and his oxygen requirements increased from 10 L and 40% FiO_2_ to 15 L and 60% FiO_2_ on venturi mask. Examination revealed a tracheal shift to the left side, a hyper‐resonant percussion note with absent breath sounds on the right side of the chest. A bedside point‐of‐care ultrasound (POCUS) showed the “bar‐code” sign and absence of sliding—a finding consistent with a right pneumothorax (Figure 2). A bedside CXR was performed, which confirmed a right‐sided TP (Figure 1B).

With a clinical and radiological diagnosis of tension pneumothorax, a 14 Fr Chest tube inserted using Seldinger technique was immediately placed and connected to under‐water seal. Over the next five days, the patient's pneumothorax improved with decreased oxygen requirement. However, due to persistent shortness of breath, oxygen requirement, and persistent bubbling, the patient's chest tube could not be removed till two months later when his pneumothorax resolved, and he started to maintain normal oxygen saturation in room air. He was discharged afterward in an asymptomatic condition, with no evidence of pneumothorax recurrence six months following the discharge.

## 3 DISCUSSION

The SARS‐CoV‐2 virus, a member of the coronavirus family, has caused over 300 million cases and more than 5.4 million deaths globally since its emergence in 2019.^1^ The B.1.1.529 variant (Omicron) is marked as the current variant of concern by WHO since November 2021 and is the cause of the recent wave of cases globally.^17^ Other than its direct and primary insult to the body, Coronavirus 2019 (COVID‐19) may result in multimodal detrimental sequela, including secondary health‐related complications, psychological effects, and socioeconomic impact at individual and global levels.^18^ Although most cases of COVID‐19 infection exhibit primarily constitutional and respiratory tract symptoms (such as fever, fatigue, myalgias, dry or productive cough, and dyspnea) similar to any other pulmonary viral infection, it can cause any other organ/system to malfunction. This includes but is not limited to ocular, neurological, gastrointestinal, olfactory, gustatory, cardiac, hepatic, renal, hematological, and cutaneous manifestations.^2^, ^19^

Concerning the pulmonary manifestations of COVID‐19, other than pneumonia and acute respiratory distress syndrome, various complications have been reported, which are not routinely seen in other types of respiratory viral infections. These include a prolonged infectious state, lung fibrosis, bullous lung disease, pleural effusion, pulmonary cysts, spontaneous pneumothorax, and pneumomediastinum, amongst others.^20^, ^21^ Most of the patients who die from COVID‐19 infection have the respiratory system as the primary organ involved.^22^ It is still unclear whether a specific mutation in the SARS‐CoV‐2 is more detrimental to the respiratory system than others, as the data concerning differences in the mechanism of infection of different variants are scarce. Given the continued pandemic amid constantly emerging mutated variants of the SARS‐CoV‐2 infection, its infectious manifestations, especially atypical and potentially fatal, remain relevant and focus of clinical interest.

Spontaneous tension pneumothorax (STP) is one of the most severe, life‐threatening complications reported multiple times with COVID‐19. STP can be primary, which mainly occurs in patients between 60 and 80 years, or secondary, which occurs in patients with pre‐existing lung disease or a significant smoking history^23^It is not unexpected for patients with chronic lung disease who develop COVID‐19 infection to have secondary STP as a complication. However, there are multiple reports of patients with only an acute respiratory insult due to COVID‐19 infection, who developed STP without any other risk factors.^5^, ^6^, ^7^, ^8^, ^9^, ^10^, ^11^, ^12^, ^13^, ^14^, ^15^, ^16^, ^24^ It is imperative to identify the signs and symptoms of STP, especially in the setting of an ongoing respiratory infection, as sometimes the clinical features can overlap and may cause diagnostic and management delays. Clinical features of STP range from common findings such as dyspnea, chest pain, tachycardia to rare features such as cyanosis, epigastric pain, and unconsciousness.^4^ STP is a clinical and radiological diagnosis. It can be diagnosed by a point‐of‐care ultrasound, chest X‐ray (CXR), or computed tomography (CT) scan. Patients on medical floors diagnosed with STP require an urgent escalation of care to intensive units for appropriate management, which is usually invasive. The usual management is a high flow oxygen supply followed by removal of the air from the pleural cavity, which can be done via emergency needle decompression with or without a chest tube insertion, depending on the clinical situation.^4^ Urgent needle decompression is the usual effective immediate treatment for STP; however, it has associated morbidity and complications. The needle is usually inserted at the second intercostal space above the rib in the midclavicular line and kept in place until a chest tube is inserted. An immediate CXR should follow this to assess the PTX resolution. When an STP results in a cardiac arrest, needle decompression is considered part of resuscitation to restore cardiac output.^25^

After encountering and managing our patient with COVID‐19 infection complicated by STP, we reviewed the available literature (from PubMed, Scopus, Cochrane Library, and Google Scholar) to identify similar cases. After reviewing 160 articles from title/abstract/keywords, and an in‐depth review of 29 articles, we finalized 12 articles (1 case series and 11 case reports, for a total of 14 patients) which fulfilled the inclusion criteria to add to this literature review (no primary lung disease, COVID‐19 infection confirmed with polymerase chain reaction test, confirmed diagnosis of STP clinically as well as radiologically). The data from these articles are presented in Table 2.

**TABLE 2:** Clinical characteristics and details of patients infected with SARS‐CoV‐2 who developed STP

Cases	Age (y), Sex, comorbid	Symptoms	Radiological findings	SPTX onset	RF for SPTX	Tx	SPTX Duration (days)	LOS (days)	Outcome
Case 1^7^	36, M, no PMH	Cough, fever, and dyspnea: 3 weeks PCP: 4 h	CXR: large left‐sided PTX, mediastinal shift, right lung consolidation	Day 1	No	ND +CD	2	4	Resolved
Case 2^6^	23, M, no PMH	Sudden onset epigastric pain and dyspnea	CXR: bilateral tension pneumothorax	Day 30	No	ND +CD	1	30	Death
Case 3^9^	83, M, no PMH	Worsening SOB and hypotension: 60/40 mmHg	CT: large left pneumothorax and ground‐glass opacities in the right lung	NA	No	CD	NA	NA	Death
Case 4^14^	62, M, T2D, HTN, PAD, CA, Previous PE	Dry cough, low grade fever, worsening SOB for 4 days, and PCP	CXR: large right‐sided pneumothorax causing mediastinal shift	Day 1	No	CD	1	1	Death
Case 5^15^	56, M, no PMH	Fever (40.2°C), nonproductive cough, SOB for 4 days	CT: Tension pneumothorax on the left side, which caused shifting the heart and trachea to the right mediastinum	Day 1	No	CD	4	4	Resolved
Case 6^12^	59, M, no PMH	Cough, fever, and SOB	CXR: Left‐sided STP	Day 11	No	ND +CD	NA	NA	Resolved
Case 7^10^	41, M, no PMH	7‐day history of SOB and fever	CXR: Large left‐sided tension pneumothorax	Day 1	No	CD	7	19	Resolved
Case 8^16^	37, M, no PMH	Right‐sided PCP, SOB of approximately 24‐h duration	CXR: Large right pneumothorax with a leftward shift of the mediastinal structures	Day 14	Rt. mid‐lung bulla	CD	5	5	Resolved
Case 9 pt. 1^5^	55, M, DM	Dyspnea, fever, sore throat cough for 1 week, Worsening of SOB 3 days	CXR‐right‐sided pneumothorax with subcutaneous emphysema	Day−10	No	CD	2	22	Resolved
Case 9 pt. 2^5^	33, M, no PMH	Fever dry cough 5 days, dyspnea 3 days	CXR showed large right‐sided pneumothorax	Day−15	Large bullae	CD	10	NA	NA
Case 9 pt. 3^5^	50, M, no PMH	Fever, dry cough, headache, dyspnea, worsening of SOB after 7 days of admission	CXR‐right‐sided pneumothorax with a mediastinal shift	Day−7	No	CD	3	NA	Resolved
Case 10^8^	64, M, DM, HTN, Dyslipidemia	Fever, dry cough, progressive SOB 2 weeks	a large right‐sided tension pneumothorax with mediastinal shift and diffuse airspace shadowing throughout the left lung, which was more pronounced peripherally	Day−1	No	CD	3	4	Resolved
Case 11^11^	55, M, no PMH	Presented with sudden onset dyspnea after 4 weeks of discharge	CT chest‐ a multiloculated right‐sided tension pneumothorax	Day−63	No	CD pleurodesis in 2nd presentation	14 days on first STP, then another 3 days for 2^nd^ STP	NA	Resolved
Case 12^13^	33, F, no PMH	Cough, SOB, myalgia 1 week	Clinically diagnosed	Day 14	Intubation	ND +CD	NA	NA	Resolved

Abbreviations: CA, cancer; CD, chest drain; CRP, C‐reactive protein; Inv, investigations; LC, lymphocyte count; NA, not available; NA, not available; ND, needle decompression; PAD, peripheral arterial disease; PCP, pleuritic chest pain; PE, pulmonary embolism; PMH, past medical history; SOB, shortness of breath; STP, spontaneous tension pneumothorax; STP, spontaneous tension pneumothorax; T2D, type II diabetes mellitus; Tx: treatment; WC, white blood cell count.

None of the patients had a prior history of PTX, which is also considered a risk factor for developing a recurrent PTX (recurrence rate is reported as high as 32%).^26^ Only one patient underwent intubation which could be argued as a reason for barotrauma‐induced STP; however, the treating physicians associated the STP to the infection itself.^13^ Two patients were found to have bullae which were not diagnosed prior to the admission with COVID‐19.^5^, ^16^ As the bullae were diagnosed after COVID‐19 infection, the treating physicians labeled them a manifestation of SARS‐CoV‐2 induced lung damage. Other than this, no other risk factors were identified in any of the other patients reported in the literature with COVID‐19 infection complicated by STP. The patients had a median age of 52.5 years (ranging from 23 to 83 years). The median age in patients with STP without COVID‐19 is reported to be the same (52.1 years) in a study of 320 patients.^27^

Interestingly, 13 out of the 14 patients (92.8%) were males. Previous studies also show a male preponderance (up to 87%), and this difference can be associated to sample size variations.^27^, ^28^ Generally, any PTX is more prevalent in men (around thrice as much), without any apparent reason.^28^ However, this difference is more pronounced in STP, especially in patients reported to have COVID‐19 infection. 78.5% (n=11) of the patients did not have any baseline comorbid condition, including prior lung disease. Manifestations of STP in this patient group were similar to the standard presentations, including fever, cough, chest pain, and shortness of breath in most of the cases. However, these symptoms are identical to those secondary to the COVID‐19 infection itself in the absence of STP. This presents a diagnostic challenge. Worsening of the same symptoms present at presentation with COVID‐19 was reported in many cases, which is a diagnostic clue and a signal toward possible STP.

Another interesting point is the onset of STP from the start of COVID‐19 infection, which seems to vary broadly. Five patients (35%) presented initially with STP, whereas it was reported to develop as late as Day 63 of COVID‐19 infection. The patient who developed STP at day 63 had two episodes. For the first one, he was managed with a chest drain. However, eventually, pleurodesis was done on the second episode.^11^ This was the only patient where STP was recurrent.

Out of 14 patients, three died (2 due to worsening STP and respiratory distress and one due to cardiac arrest as a complication of STP).^6^, ^7^, ^9^ No specific pattern was found in the three patients who died concerning age, presenting symptoms, duration of STP, and its management. Due to scarce data in the literature, analyzing the factors related to mortality is difficult, hence requiring urgent prospectively designed studies to delineate the prognostic factors in this patient population.

The mortality of patients with STP in the presence of COVID‐19 infection is much higher than that reported without COVID‐19 infection (21.4% vs. 3%–7%).^27^, ^29^ This high mortality rate in patients, the majority of whom had no prior medical condition, is concerning and requires further research to identify factors associated with mortality.

As new data are evolving and much is still unknown about how, to what extent, and in what ways SARS‐CoV‐2 can infect the human body, it is imperative to keep an eye on patients who present with uncommon complications, especially with high mortality. The global mortality rate of COVID‐19 infection is around 2%, whereas our literature review of patients with COVID‐19 infection who developed STP highlights that the mortality in these patients can be much higher (approximately 21.4%). Although there can be various confounders here (such as small sample size, varying degrees of COVID‐19 severity amongst others), presence of an increasing number of cases of STP in COVID‐19 patients, without any other identifiable reason, is a clinical scenario which must be addressed. It is imperative to study STP in COVID‐19 on a larger population with prospectively designed studies to identify the actual prevalence and mortality rates. These studies will also help generate other valuable information concerning the severity of lung involvement in such patients, duration of the pneumothorax and management of COVID‐19 in the context of STP. STP is a reversible cause of shortness of breath; however, if left untreated, it can be fatal.^12^ Hence, all efforts should be placed to timely diagnose and manage these cases amid a continued pandemic.

## 4 CONCLUSION

Spontaneous tension pneumothorax should be included in the differential diagnosis of COVID‐19 infected patients who have worsening symptoms or develop new respiratory symptoms during their hospital course. The management should be the same as any STP; however, an early diagnosis and prompt treatment may help reduce the seemingly higher than expected mortality with this complication of SARS‐CoV‐2 infection. More extensive studies are required to identify true prevalence and mortality rates as well as the factors associated with occurrence, as well as mortality in this patient population.

## AUTHOR CONTRIBUTIONS

Zohaib Yousaf and Talat Saeed Chughtai case identification and eligibility assessment. Fateen Ata, Maya Omran, Dore Chikkahanasoge Ananthegowda, and Abdullah Arshad involved in data collection. Fateen Ata, Zohaib Yousaf, Adeel Ahmed Khan, and Rana Farsakoury involved in initial manuscript writing. Mohamad Khatib and Talat Saeed Chughtai performed critical review of manuscript and revisions. Talat Saeed Chughtai supervised the manuscript. All authors involved in literature review and reviewed and approved the final manuscript.

## CONFLICT OF INTEREST

The authors declare that they have no competing interests.

## ETHICAL APPROVAL

Ethics approval for the case was obtained from Medical Research Center (MRC) Qatar (MRC‐04–21–268).

## CONSENT FOR PARTICIPATION AND PUBLICATION

Written informed consent was obtained from the patient for data and accompanying images prior to submission of this manuscript.

## CONSENT

Written informed consent was obtained from the patient for publication of this case report and accompanying images.

## Data Availability

Data sharing is not applicable.
